# Neurite Outgrowth-Promoting Activity of Compounds in PC12 Cells from Sunflower Seeds

**DOI:** 10.3390/molecules25204748

**Published:** 2020-10-16

**Authors:** Takeru Koga, Takaiku Sakamoto, Eiji Sakuradani, Akihiro Tai

**Affiliations:** 1 Graduate School of Advanced Technology and Science, Tokushima University, 2-1 Minamijosanjima-cho, Tokushima 770-8506, Japan; c502044005@tokushima-u.ac.jp; 2 Graduate School of Technology, Industrial and Social Sciences, Tokushima University, 2-1 Minamijosanjima-cho, Tokushima 770-8513, Japan; sakamoto.takaiku@tokushima-u.ac.jp (T.S.); sakuradani.eiji@tokushima-u.ac.jp (E.S.)

**Keywords:** Alzheimer’s disease, sunflower seed, NGF, β-sitosterol, neurite outgrowth-promoting activity

## Abstract

In the current super-aging society, the establishment of methods for prevention and treatment of Alzheimer’s disease (AD) is an urgent task. One of the causes of AD is thought to be a decrease in the revel of nerve growth factor (NGF) in the brain. Compounds showing NGF-mimicking activity and NGF-enhancing activity have been examined as possible agents for improving symptoms. In the present study, sunflower seed extract was found to have neurite outgrowth-promoting activity, which is an NGF-enhancing activity, in PC12 cells. To investigate neurite outgrowth-promoting compounds from sunflower seed extract, bioassay-guided purification was carried out. The purified active fraction was obtained by liquid-liquid partition followed by some column chromatographies. Proton nuclear magnetic resonance and gas chromatography-mass spectrometry analyses of the purified active fraction indicated that the fraction was a mixture of β-sitosterol, stigmasterol and campesterol, with β-sitosterol being the main component. Neurite outgrowth-promoting activities of β-sitosterol, stigmasterol, campesterol and cholesterol were evaluated in PC12 cells. β-Sitosterol and stigmasterol showed the strongest activity of the four sterol compounds (β-sitosterol ≈ stigmasterol > campesterol > cholesterol), and cholesterol did not show any activity. The results indicated that β-sitosterol was the major component responsible for the neurite outgrowth-promoting activity of sunflower seeds. Results of immunostaining also showed that promotion by β-sitosterol of neurite formation induced by NGF was accompanied by neurofilament expression. β-Sitosterol, which showed NGF-enhancing activity, might be a candidate ingredient in food for prevention of AD.

## 1. Introduction

Alzheimer’s disease (AD) is a global health challenge, and the number of patients with AD has been increasing worldwide [1]. It is expected that the number of AD patients will continue to increase with the aging of the world population, and it is estimated that the number will reach 115 million by 2050 [1]. Patients with AD have reduced activity of acetylcholine, deposition of amyloid β and a decrease in the revel of nerve growth factor (NGF) in the brain, but the exact cause of AD has not been clarified [2]. Acetylcholinesterase inhibitors are used for relieving symptoms in AD patients. However, the effects of treatment with these inhibitors are transient and moderate [3]. Therefore, new radical treatments for AD have been needed.

It is thought that NGF is the key to amelioration of cognitive function since NGF supports survival, differentiation and maintenance of neurons [4]. It was reported that hippocampal NGF levels were reduced in aged rats [5]. It was also reported that continuous infusion of NGF to the brain can improve retention of spatial memory in behaviorally impaired elderly rats [6]. The results of those studies suggest that NGF administration might be effective for improving the condition of AD patients. However, the application of NGF as a medicine for AD is difficult because NGF cannot pass through the blood–brain barrier (BBB) due to its physicochemical properties [7,8]. To solve this problem, considerable efforts have been made to find low-molecular-weight and lipophilic compounds that have neurotrophic properties similar to those of NGF and can pass through the BBB [8]. It has been reported that lindersin B, a lipophilic triterpenoid that was isolated from *Lindernia crustacea*, induced neurite outgrowth in PC12 cells [9]. It was also reported that isofuranodiene, which is the major constituent of essential oil of wild celery (*Smyrnium olusatrum* L., Apiaceae), has neurite outgrowth-promoting activity in the presence of NGF in PC12 cells [10]. These compounds with NGF-mimicking or NGF-enhancing effects are likely to be able to pass the BBB since they are low-molecular-weight and lipophilic compounds. Hence, it is speculated that the use of low-molecular-weight and lipophilic compounds with NGF-mimicking or NGF-enhancing activity is an effective approach for AD treatment.

Plant seeds are rich in oil and are used as foods worldwide. Sunflower (*Helianthus annuus* L.) seeds are nutritious and are used as cooking oil and as roasted or salted snacks. It was reported that administration of oil obtained from sunflower seeds increased the amount of phosphatidylcholine in the rat brain [11]. Phosphatidylcholine is used as a material for myelin-supporting synapse formation [12]. Also, acetylcholine, a neurotransmitter, is synthesized from phosphatidylcholine to help transmission of information and improvement of memory in the brain [13]. Phosphatidylcholine is synthesized in neuronal cells during neurite outgrowth [14,15], and a lack of phosphatidylcholine induces apoptosis in PC12 cells [16,17]. Accordingly, it has been considered that ingestion of phosphatidylcholine would improve cognitive function and prevent dementia [18]. Despite reports of these effects, NGF-mimicking or NGF-enhancing activity of sunflower seeds has not been investigated.

This study aims to isolate and identify compounds showing neurite outgrowth-promoting activity, which is an NGF-enhancing effect, from sunflower seed extract. Active compounds were purified by activity-guided fractionation and analyzed by proton nuclear magnetic resonance (^1^H-NMR) and gas chromatography-mass spectrometry (GC-MS). Herein, the purification and identification of compounds showing neurite outgrowth-promoting activity, and the structure–activity relationship of related compounds, are reported. In addition, it is shown that the promoting activity of the most potent compound is neurite formation with expression of neurofilaments.

## 2. Results and Discussion

### 2.1. Isolation of β-Sitosterol from Sunflower Seeds

Sunflower seeds (164.69 g) were extracted with acetone-methanol (8/2, *v*/*v*), and the extract showed neurite outgrowth-promoting activity in the presence of NGF or dibutyryl cyclic AMP (Bt_2_cAMP) in PC12 cells (Figures S1 and S2 in the Supplementary Materials, published with this article online). NGF binds to the tropomyosin receptor kinase A receptor and extends neurites via a signaling cascade that includes extracellular signal-regulated kinase (ERK) [19,20]. As another signal pathway, NGF increases intracellular cAMP concentration, which induces neurite formation [21]. Bt_2_cAMP, which is a membrane-permeable cAMP derivative, is metabolized intracellularly to cAMP and shows a neurite formation effect [22]. Therefore, in order to efficiently evaluate the neurite outgrowth-promoting activity in a short time and to purify the active compounds, Bt_2_cAMP was applied as a neurite formation inducer in PC12 cells.

**Figure 1:**
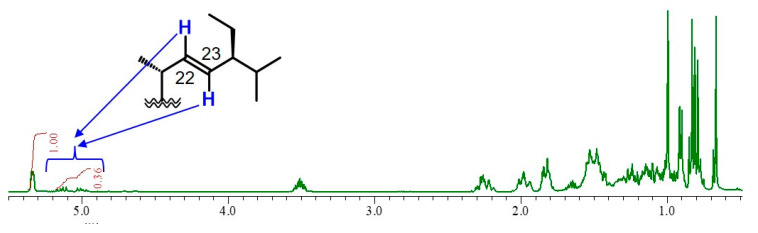
^1^H-NMR spectrum of the purified fraction. The ^1^H-NMR spectrum showed that the purified fraction was sterol. The arrows show two characteristic signals of trans-protons attached to the C22–C23 double bond of stigmasterol.

The sunflower seed extract was separated between hexane and methanol after concentration, and then the hexane layer was chromatographed on a Wakogel C-200 column twice and on a TOYOPEARL HW-40F, by active-guided fractionation, to obtain a white powder (123.1 mg). It was thought that the white powder was a sterol compound since the ^1^H-NMR data of the white powder were similar to those of β-sitosterol in previous reports [23,24,25] (Figure 1). However, the ^1^H-NMR spectrum of the purified compound contained small signals on δ 4.9–5.2, which were not observed in the ^1^H-NMR spectrum of β-sitosterol. The signals at δ 5.00 and δ 5.14 seemed to be consistent with two characteristic signals of trans-olefinic protons at the C22 and C23 positions of stigmasterol. The integral value of the signals that are thought to be derived from two protons at the C22–C23 positions of stigmasterol was 0.36, whereas the integral value of the signal of a proton at the C6 position on sterols was 1.00 (Figure 1). The ^1^H-NMR data suggested that the purified fraction was a mixture of β-sitosterol and stigmasterol, which contained β-sitosterol as the main compound.

**Figure 2:**
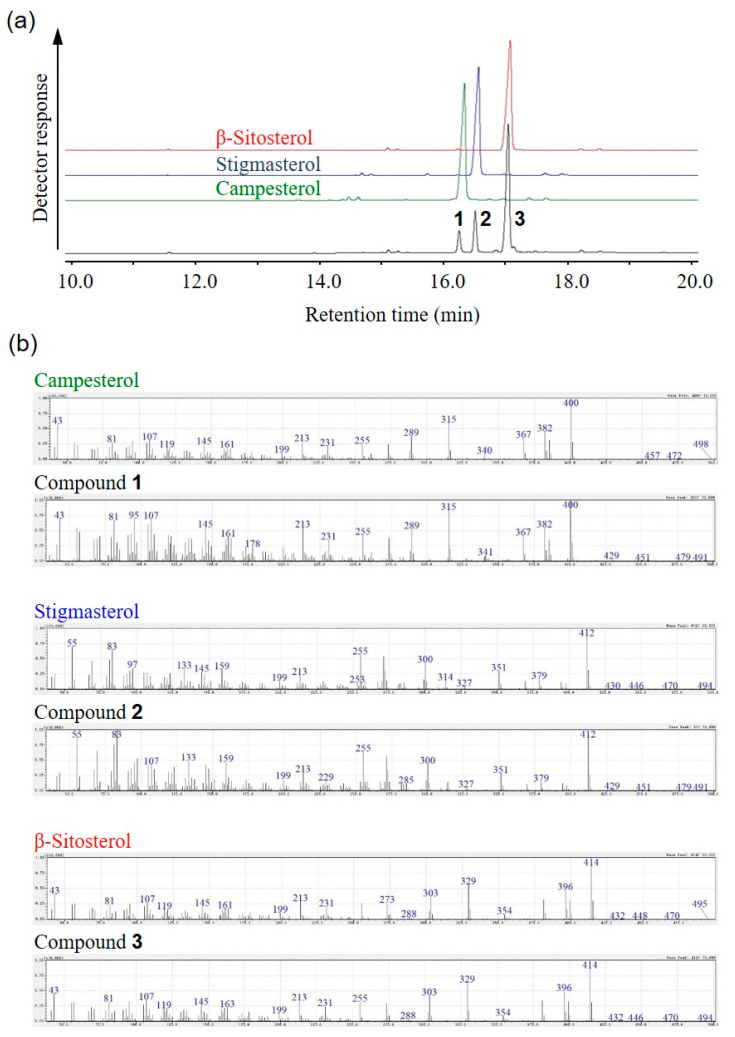
GC-MS chromatograms of the purified fraction and standards of campesterol, stigmasterol and β-sitosterol. (**a**) Total ion chromatograms of campesterol, stigmasterol, β-sitosterol and purified fraction. (**b**) GC-MS fragment ions of the purified fraction and standards of campesterol, stigmasterol and β-sitosterol.

To clarify the ingredients of the fraction, the fraction showing neurite outgrowth-promoting activity in the presence of Bt_2_cAMP in PC12 cells was analyzed by GC-MS. The GC-MS data indicated that the active fraction was a mixture of three compounds (compounds **1**, **2** and **3**) and that the retention times of compounds were approximately 16.2, 16.5 and 17 min, respectively (Figure 2a). The retention times of compounds **1**, **2** and **3** were in good accordance with those of the standards of campesterol, stigmasterol and β-sitosterol, respectively (Figure 2a). In addition, the molecular ions and fragment ions of compounds **1**, **2** and **3** agreed fairly well with those of the standards of campesterol, stigmasterol and β-sitosterol, respectively (Figure 2b). GC-MS also showed that the peak-area ratio of campesterol, stigmasterol and β-sitosterol was about 1:2:7 (Figure 2a). Therefore, these results showed that the fraction was a mixture of β-sitosterol, stigmasterol and campesterol, the main component of which is β-sitosterol.

The ^1^H-NMR and GC-MS data allowed unequivocal identification of these compounds, β-sitosterol, stigmasterol and campesterol, in the purified active fraction. The identification of these compounds was supported by a report of the material composition in sunflower oil [26]. β-Sitosterol, stigmasterol and campesterol are well-known compounds, and it was previously reported that these compounds, which are phytosterols, are abundant in plants, fruits and seeds [27,28,29]. It was reported that a phytosterol was effective for lowering low density lipoprotein cholesterol in patients with type 2 diabetes [30]. It was also reported that the amount of phytosterols intake was associated with improved survival in hypertensive rats [31]. However, neurite outgrowth-promoting activities of these compounds have not been reported.

### 2.2. Promoting Activity of Sterols in Neurite Formation Induced by Bt_2_cAMP in PC12 Cells

**Figure 3:**
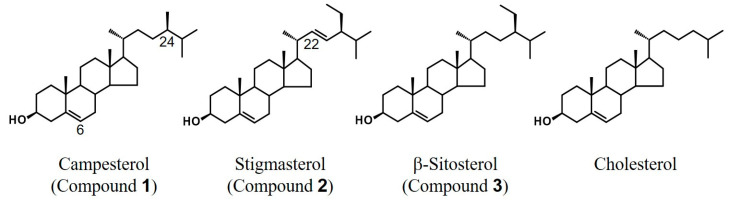
Structures of purified compounds and cholesterol.

A mixture of β-sitosterol, stigmasterol and campesterol, which was composed mainly of β-sitosterol, was purified from sunflower seed extract as neurite outgrowth-promoting active compounds in the presence of Bt_2_cAMP in PC12 cells. The structures of β-sitosterol and campesterol have an ethyl group and a methyl group respectively, at the C24 position of the cholesterol skeleton (Figure 3). Stigmasterol has an unsaturated double bond at the C22 position of the β-sitosterol structure (Figure 3). It is known that sunflower oil includes a small amount of cholesterol in addition to β-sitosterol, stigmasterol and campesterol [26]. Hence, to compare the strengths of the neurite outgrowth-promoting activities of β-sitosterol, stigmasterol, campesterol and cholesterol, four sterols were evaluated in the presence of Bt_2_cAMP in PC12 cells.

**Figure 4:**
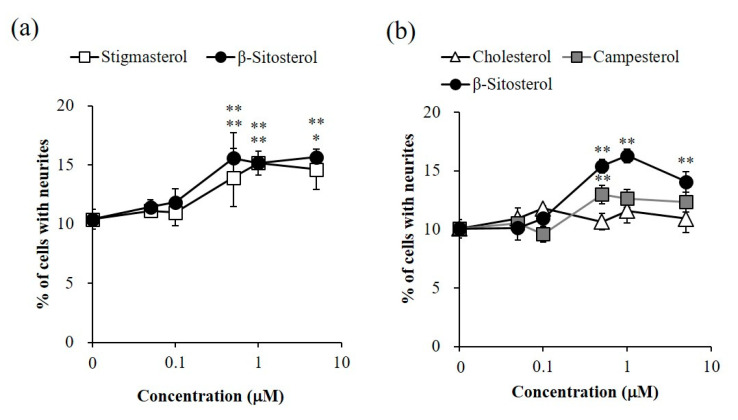
Neurite outgrowth-promoting activities of sterols in the presence of Bt_2_cAMP in PC12 cells. (**a**) Promotion by β-sitosterol and stigmasterol of neurite formation induced by Bt_2_cAMP in PC12 cells. (**b**) Promotion by β-sitosterol, campesterol and cholesterol of neurite formation induced by Bt_2_cAMP in PC12 cells. PC12 cells were plated at 4.0 × 10^3^ cells/well and cultured with the samples at 0.05–5 μM in the presence of 0.5 mM of Bt_2_cAMP. The extent of neurite outgrowth was measured at 24 h and is expressed as the mean percentage of 300–400 cells. The data represent means ± standard deviation (SD) from three independent experiments. * *p* < 0.05, ** *p* < 0.01 (Dunnett’s test) as compared with control (0.5 mM Bt_2_cAMP only).

The intensities of neurite outgrowth-promoting activities of β-sitosterol and stigmasterol, and those of β-sitosterol, campesterol and cholesterol, were compared. The activities of β-sitosterol and stigmasterol were significant at 0.5–5 μM, and the intensities of the activities of β-sitosterol and stigmasterol were similar (Figure 4a). Campesterol showed significant activity at 0.5 μM, but the activity of campesterol was weaker than that of β-sitosterol (Figure 4b). Cholesterol did not show activity at any of the tested concentrations (Figure 4b). Evaluation of the activities of the four sterols, β-sitosterol, stigmasterol, campesterol and cholesterol, revealed relationships between the structures and active intensities of the sterols. β-Sitosterol and stigmasterol showed similar significant activities (Figure 4a), indicating that introduction of a carbon–carbon double bond to the C22–C23 position of β-sitosterol did not affect its activity. Moreover, the intensities of the activities of β-sitosterol, campesterol and cholesterol were in the order of β-sitosterol, campesterol and cholesterol (Figure 4b). Therefore, it was thought that the activity was enhanced depending on the length of the side chain attaching to the C24 position of cholesterol. The results indicated that β-sitosterol and stigmasterol had the strongest neurite outgrowth-promoting activities of the four sterols evaluated and that the intensities of activities of β-sitosterol and stigmasterol were similar. Additionally, the active purified fraction, which was mixture of β-sitosteol, stigmasterol and campesterol, was revealed by ^1^H-NMR and GC-MS analyses to have β-sitosterol as the main component (Figure 1 and Figure 2). Therefore, the results suggested that β-sitosterol was the main compound with neurite outgrowth-promoting activity in the fraction purified from sunflower seed extract.

β-Sitosterol, which is the main compound showing neurite outgrowth-promoting activity in sunflower seed extract, is one of the well-known plant sterols and is found in avocados, soybeans and corn [32,33]. It was reported that β-sitosterol has cholesterol metabolic action, antibacterial action and anticancer action [34,35,36]. However, neurite outgrowth-promoting activity of β-sitosterol has not been reported.

### 2.3. Promoting Activity of β-Sitosterol on Neurite Formation Induced by NGF in PC12 Cells

**Figure 5:**
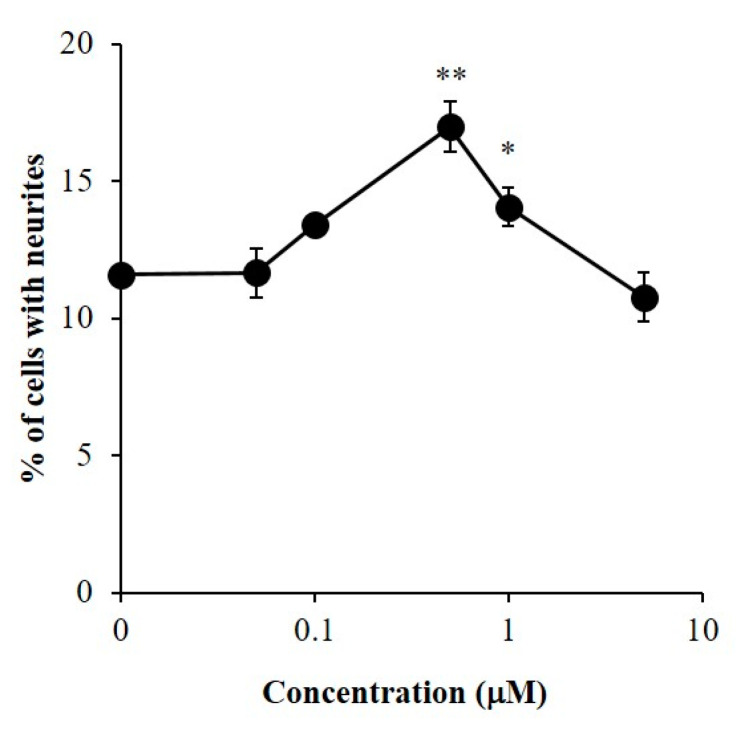
Neurite outgrowth-promoting activity of β-sitosterol in the presence of NGF in PC12 cells. PC12 cells were plated at 2.0 × 10^3^ cells/well and cultured with the samples at 0.05–5 μM in the presence of 10 ng/mL of NGF. The extent of neurite outgrowth was measured at 48 h and is expressed as the mean percentage of 300–400 cells. The data represent means ± SD from three independent experiments. * *p* < 0.05, ** *p* < 0.01 (Dunnett’s test) as compared with control (10 ng/mL of NGF only).

The NGF-enhancing effect of β-sitosterol was shown by confirming the promoting activity of β-sitosterol on neurite formation induced by NGF in PC12 cells. In a bioassay, β-sitosterol showed significant neurite outgrowth-promoting activities at 0.5 and 1 μM in the presence of NGF in PC12 cells (Figure 5). It was proved that β-sitosterol can promote neurite outgrowth induced by NGF as well as Bt_2_cAMP.

**Figure 6:**
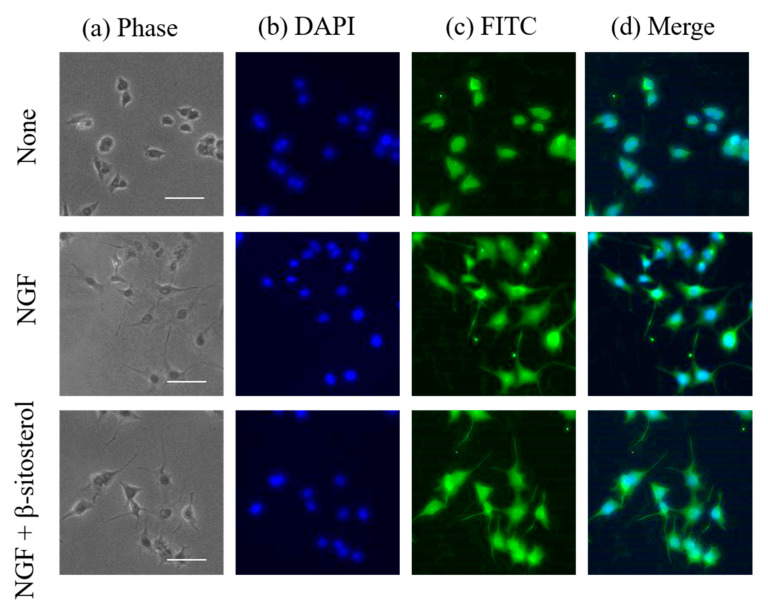
Immunostaining of neurofilaments of PC12 cells with differentiated neurites stimulated by β-sitosterol in the presence of NGF. PC12 cells were incubated for 48 h without or with NGF (10 ng/mL) and β-sitosterol (0.5 μM) or with NGF (10 ng/mL) alone. Immunostaining was performed with rabbit anti-neurofilament antibody, followed by treatment with fluorescein isothiocyanate-conjugated goat anti-rabbit IgG. The nuclei of PC12 cells were treated with 4′,6-diamidino-2-phenylindole (blue) and examined under a fluorescence microscope. (**a**) Phase contrast, (**b**) DAPI-treated nuclei, (**c**) green fluorescence for FITC-treated neurofilaments and (**d**) merged images. Scale bar = 50 μm.

Next, neuronal differentiation in NGF-induced neurite formation enhanced by β-sitosterol in PC12 cells was observed by immunocytochemical analysis. Neurofilaments are type IV intermediated filaments that are expressed specifically in nerve cells and are involved in the maintenance of nerve thickness. β-Sitosterol showed significant activities at 0.5 and 1 μM, and the activity of β-sitosterol at 0.5 μM was stronger than that at 1 μM (Figure 5). Expression of neurotrophy of PC12 cells stimulated by 0.5 μM of β-sitosterol in the presence of NGF was assessed by using a fluorescence microscope (Figure 6). Neurite outgrowth in PC12 cells and expression of neurofilaments at the cell body by stimulation with β-sitosterol were confirmed by immunocytochemical analysis. The results suggested that neuronal differentiation induced by NGF in PC12 cells was promoted by stimulation with β-sitosterol. To our knowledge, this is the first report showing a novel function of β-sitosterol as an NGF action enhancer. Acetylcholinesterase inhibitory activity of β-sitosterol was previously reported [37]. It was also shown that β-sitosterol can prevent the accumulation of amyloid peptide, which is thought to be one of the causes of AD [38]. It was also reported that β-sitosterol can pass through the BBB and accumulate in the murine brain cells [39]. In addition to these reports, the new finding of neurite outgrowth-promoting activity of β-sitosterol in this study indicates that β-sitosterol is a more effective candidate for the prevention of AD.

## 3. Materials and Methods

### 3.1. Materials

Sunflower seeds (Sunny Kids) were purchased from Sakata Seed Corporation (Kanagawa, Japan). Acetone, methanol, ethanol, hexane, ethyl acetate (EtOAc), chloroform-*d* (CDCl_3_), giemsa stain solution, 25% glutaraldehyde solution, polyoxyethylen (20) sorbitan monolaurate (Tween 20) and cholesterol were obtained from FUJIFILM Wako Pure Chemical Corporation (Osaka, Japan). RPMI-1640 medium, dibutyryl cyclic AMP (Bt_2_cAMP), anti-Rabbit IgG (whole molecular)-fluorecein isothiocyanate (FITC) antibody produced in a goat (Batch No. 073M4789), Triton X-100 and anti-neurofilament 200 IgG fraction of antiserum (Lot. 091K4832) were purchased from Sigma-Aldrich Japan (Tokyo, Japan). Penicillin-streptomycin mixed solution, sodium dihydrogenphosphate dihydrate, di-sodium hydrogenphosphate, paraformaldehyde and bovine serum albumin (BSA) were obtained from Nacalai Tesque (Kyoto, Japan). Fetal bovine serum (FBS) (Lot. 42F9155K) and horse serum (HS) (Lot. 1517707) were obtained from Gibco (Waltham, MA, USA). Cellmatrix type I-P (collagen) was purchased from Nitta Gelatine (Osaka, Japan). Ninety-six-well plates (167008, Nunc) were obtained from Thermo Fisher Scientific K.K. (Tokyo, Japan). β-Sitosterol, stigmasterol and campesterol were purchased from Tama Biochemical Co., Ltd. (Tokyo, Japan). 4′,6-Diamino-2-phenylindole (DAPI)-Fluoromount-G was purchased from Cosmo Bio Co., Ltd. (Tokyo, Japan). γγβ-NGF (Lot. XF8314021) (NGF) was purchased from R&D SYSTEMS (USA). Wakogel C-200 (FUJIFILM Wako Pure Chemical Corporation, Osaka, Japan) and TOYOPEARL HW-40F (Tosoh Corporation, Tokyo, Japan) were used for column chromatography. A ^1^H-NMR spectrum was obtained on a JEOL NMR system (ECZ400) 400 MHz instrument. Gas chromatography-mass spectrometry (GC-MS) was performed using a GC-2030 Nextis (Shimadzu Corporation, Kyoto, Japan) with a GCMS-QP2020 NX (Shimadzu Corporation), an AOC-20i Plus automatic injector (Shimadzu Corporation) and an HP-5MS capillary column of 30 m in length, 0.25 mm in internal diameter, and 0.25 μm in film thickness (Agilent Technologies, Santa Clara, CA, USA). Helium 99.999% was used as the carrier gas at a constant flow rate of 3 mL/min.

### 3.2. Isolation of β-Sitosterol from Sunflower Seed Extract

Sunflower seeds (887.28 g) were milled and extracted with 4436.4 mL of acetone-methanol (8/2, *v*/*v*). The acetone-methanol extract was then evaporated to dryness to obtain a crude extract (164.69 g, oil wt.). The extract was dissolved in 700 mL of methanol and partitioned with 700 mL of hexane three times. The separated layers were then evaporated to dryness (hexane layer: 159.25 g, methanol layer: 7.64 g). Next, the hexane layer from the extract of sunflower seeds was chromatographed on Wakogel C-200 (Ø 7.0 × 29.2 cm) and eluted with a stepwise hexane/EtOAc gradient (10/0, 9.5/0.5, 9/1, 8/2, 7/3 each 1680 mL and 5/5, *v*/*v*, 2240 mL). The 80% hexane/EtOAc eluents were combined, and then the active combined fractions (1.62 g) were chromatographed on Wakogel C-200 (Ø 3.2 × 29.0 cm) again and eluted with a stepwise hexane/EtOAc gradient (10/0, 9.5/0.5, 9/1, 8.5/1.5, 8/2, 7.5/2.5, 7/3, *v*/*v*, 280 mL each). The eluted fractions in hexane/EtOAc (8.5/1.5, *v*/*v*) were combined, and the combined fractions showed significant activity. Moreover, the combined fractions (416.6 mg) were purified by using TOYOPEARL HW-40F (Ø 2.5 × 82.5 cm) with 810 mL of EtOH to obtain 100 fractions. Fractions 33–35 (123.1 mg), which showed significant activity, were isolated as a white powder. The purified active fraction from sunflower seed extract was analyzed by ^1^H-NMR spectroscopy (CDCl_3_, 400 MHz). The chemical structures of the compounds in the fraction were determined by GC-MS analysis. The purified fraction and sterol specimens (campesterol, stigmasterol and β-sitosterol) were dissolved in chloroform to a concentration of 1 mg/mL each and used for GC-MS analysis. The inlet temperature was kept at 320 °C and the injection volume was 1 μL with a splitless time of 1.0 min. The initial column temperature was 180 °C and the temperature was kept at 180 °C for 1.0 min. Then, the temperature was ramped up to 320 °C at a rate of 10 °C/min. The total run time was 30 min. The interface temperature was 260 °C and the ion trap temperature was 200 °C. The ion trap was operated with electron ionization (EI) at 70 eV in scan mode between ions of *m*/*z* 35 and 500 with a solvent delay of 1.1 min.

### 3.3. Neurite Outgrowth-Promoting Activity

PC12 cells were purchased from RIKEN BRC Cell Bank (Tsukuba, Japan). PC12 cells were grown in RPMI-1640 supplemented with 10% HS, 5% FBS, 100 U/mL penicillin G and 100 μg/mL streptomycin at 37 °C in a humidified atmosphere of 95% air/5% CO_2_. Samples were dissolved in DMSO (final concentration: 0.5%), and then diluted with the basal culture medium. PC12 cells from stock cultures were suspended in the medium and plated at 4.0 × 10^3^ cells/90 μL/well (for evaluation in the presence of Bt_2_cAMP) or 2.0 × 10^3^ cells/90 μL/well (for evaluation in the presence of NGF) in 96-well plates coated with collagen and incubated in a humidified atmosphere of 5% CO_2_ at 37 °C. After 24 h, 5 μL of Bt_2_cAMP at 10 mM (final concentration: 0.5 mM) or NGF at 200 ng/mL (final concentration: 10 ng/mL) and 5 μL of each sample (final concentration of sterol compounds: 0.05–5 μM) or control (medium only) were added to the culture medium. At 24 h after the addition of Bt_2_cAMP and samples, or at 48 h after the addition of NGF and samples, the medium was aspirated, and PC12 cells were fixed with phosphate buffer (pH 7.2, 100 mM) containing 1% glutaraldehyde solution and stained by giemsa stain solution. Then, the 96-well plates were washed twice with Milli-Q grade water. The number of cells bearing neurites longer than twice the diameter of one cell body after treatment was divided by the total number of cells, which amounted to 300–400 cells per well.

### 3.4. Immunocytochemical Analysis

PC12 cells from stock cultures were suspended in the medium and plated at 2.0 × 10^3^ cells/90 μL/well in 96-well plates that were pre-coated with collagen and incubated in a humidified atmosphere of 5% CO_2_ at 37 °C for 24 h. Then, 5 μL of NGF at 200 ng/mL and 5 μL of β-sitosterol at 10 μM were added to the culture medium. The cells were incubated in a humidified atmosphere of 5% CO_2_ at 37 °C for 48 h. Then, the cells cultured on the 96-well plate were fixed with 100 μL of 4% paraformaldehyde in phosphate buffer (pH 7.4, 100 mM) for 30 min at room temperature. After 4% paraformaldehyde had been removed and the cells had been washed three times with phosphate buffer saline (PBS(-)) (pH 7.4), the fixed cells were permeabilized with 100 μL of 0.4% Triton X-100/PBS(-) for 10 min at room temperature. The permeabilized cells were blocked with 100 μL of 2.5% BSA/PBS(-) for 1 h at room temperature. After removal of 2.5% BSA/PBS(-), 100 μL of anti-neurofilament 200 IgG fraction of antiserum (primary antibody) in 2.5% BSA/PBS(-) (1/80, *v*/*v*) was added to each well and incubated for 2 h at room temperature. After the primary antibody solution had been removed and the cells had been washed three times with PBST (PBS(-) containing 0.05% Tween 20), 100 μL of anti-Rabbit IgG (whole molecule)-FITC antibody produced in a goat (secondary antibody) in blocking solution (1/80, *v*/*v*) was added to each well and incubated for 1 h at room temperature in the dark. Then, the secondary antibody solution was removed, and the cells were washed three times with PBST. Then, 100 μL of DAPI-Fluoromount-G/PBS(-) (8/2, *v*/*v*) was added and incubated for 10 min, and the PC12 cells were washed once with PBST and air-dried. The samples were observed by using a fluorescence microscope (BZ-X700, KEYENCE) (DAPI: Ex., 360/40 nm; Em., 460/50 nm; FITC: Ex., 470/40 nm; Em., 525/50 nm).

## 4. Conclusions

A fraction showing neurite outgrowth-promoting activity in the presence of Bt_2_cAMP in PC12 cells was purified from sunflower seed extract. It was clarified by ^1^H-NMR and GC-MS analyses that the active fraction was a mixture of β-sitosterol, stigmasterol and campesterol, with β-sitosterol being the main component (Figure 1 and Figure 2). Next, the neurite outgrowth-promoting activities of β-sitosterol, stigmasterol, campesterol and cholesterol in the presence of Bt_2_cAMP were compared. β-Sitosterol and stigmasterol showed the strongest activity among the four sterols (β-sitosterol ≈ stigmasterol > campesterol > cholesterol), and cholesterol did not show any activity (Figure 4). Since the purified fraction from sunflower seed extract contained β-sitosterol as the main component, and β-sitosterol as well as stigmasterol showed the strongest activity of the four sterols that were evaluated, it was speculated that β-sitosterol was the main component showing neurite outgrowth-promoting activity in the purified fraction. In addition, the relationships between the structures and activities of the four sterols revealed that introduction of a carbon–carbon double bond at the C22–C23 position of β-sitosterol did not affect the activity, and that the length of the side chain at the C24 position of sterols was important for the activity. Moreover, β-sitosterol exhibited promoting activity for neurite formation with neurofilament expression induced by NGF in PC12 cells (Figure 6). A previous study showing that β-sitosterol can penetrate the BBB indicates the possibility of improving AD by ingestion of β-sitosterol. Our results provide evidence that β-sitosterol is a candidate substance in food for the prevention of AD.

## Supplementary Materials

Figure S1: Neurite outgrowth-promoting activity of sunflower seed extract in the presence of NGF in PC12 cells. Figure S2: Neurite outgrowth-promoting activity of sunflower seed extract in the presence of Bt_2_cAMP in PC12 cells.

## References

[1] Wortmann M. (2012). Dementia: A global health priority—Highlights from an ADI and World Health Organization report. Alzheimer’s Res. Ther..

[2] Vasic V., Barth K., Schmidt M.H.H. (2019). Neurodegeneration and neuro-regeneration-Alzheimer’s disease and stem cell therapy. Int. J. Mol. Sci..

[3] Douchamps V., Mathis C. (2017). A second wind for the cholinergic system in Alzheimer’s therapy. Behav. Pharmacol..

[4] Mitra S., Behbahani H., Eriksdotter M. (2019). Innovative therapy for Alzheimer’s disease-with focus on biodelivery of NGF. Flont. Neurosci..

[5] Lärkfors L., Ebendal T., Whittemore S.R., Persson H., Hoffer B., Olson L. (1987). Decreased level of nerve growth factor (NGF) and its messenger RNA in the aged rat brain. Brain Res..

[6] Fischer W., Wictorin K., Björklund A., Williams L.R., Varon S., Gage F.H. (1987). Amelioration of cholinergic neuron atrophy and spatial memory impairment in aged rats by nerve growth factor. Nature.

[7] Poduslo J.F., Curran G.L. (1996). Permeability at the blood-brain and blood-nerve barriers of the neurotrophic factors: NGF, CNTF, NT-3, BDNF. Mol. Brain Res..

[8] Connor B., Dragunow M. (1998). The role of neuronal growth factors in neurodegenerative disorders of the human brain. Brain Res. Rev..

[9] Cheng L., Ye Y., Xiang L., Osada H., Qi J. (2017). Lindersin B from *Lindernia crustacea* induces neuritogenesis by activation of tyrosine kinase A/phosphatidylinositol 3 kinase/extracellular signal-regulated kinase signaling pathway. Phytomedicine.

[10] Mustafa A.M., Maggi F., Papa F., Kaya E., Dikmen M., Öztürk Y. (2016). Isofuranodiene: A neuritogenic compound isolated from wild celery (*Smyrnium olusatrum* L., Apiaceae). Food Chem..

[11] Foot M., Cruz T.F., Clandinin M.T. (1982). Influence of dietary fat on the lipid composition of rat brain synaptosomal and microsomal membranes. Biochem. J..

[12] Wurtman R.J. (2008). Synapse formation and cognitive brain development: Effect of docosahexaenoic acid and other dietary constituents. Metabolism.

[13] Chung S.Y., Moriyama T., Uezu E., Uezu K., Hirata R., Yohena N., Masuda Y., Kokubu T., Yamamoto S. (1995). Administration of phosphatidylcholine increases brain acetylcholine concentration and improves memory in mice with dementia. J. Nutr..

[14] Carter J.M., Waite K.A., Campenot R.B., Vance J.E., Vance D.E. (2003). Enhanced expression and activation of CTP: Phosphocholine cytidylyltransferase β2 during neurite outgrowth. J. Biol. Chem..

[15] Araki W., Wurtman R.J. (1997). Control of membrane phosphatidylcholine biosynthesis by diacylglycerol levels in neuronal cells undergoing neurite outgrowth. Proc. Natl. Acad. Sci. USA.

[16] Yen C.L., Mar M.H., Meeker R.B., Fernandes A., Zeisel S.H. (2001). Choline deficiency induces apoptosis in primary cultures of fetal neurons. FASEB J..

[17] de Chaves E.D., Vance D.E., Campenot R.B., Vance J.E. (1995). Axonal synthesis of phosphatidylcholine is required for normal axonal growth in rat sympathetic neurons. J. Cell Biol..

[18] Paoletti L., Elena C., Domizi P., Banchio C. (2011). Role of phosphatidylcholine during neuronal differentiation. IUBMB Life.

[19] Duan L., Hope J.M., Guo S., Ong Q., François A., Kaplan L., Scherrer G., Cui B. (2018). Optical activation of trkA signaling. ACS Synth. Biol..

[20] Vaudry D., Stork P.J., Lazarovici P., Eiden L.E. (2002). Signaling pathways for PC12 cell differentiation: Making the right connections. Science.

[21] Schulze I., Perez-Polo J.R. (1982). Nerve growth factor and cyclic AMP: Opposite effects on neuroblastoma-substrate adhesion. J. Neurosci. Res..

[22] Gunning P.W., Landreth G.E., Bothwell M.A., Shooter E.M. (1981). Differential and synergistic actions of nerve growth factor and cyclic AMP in PC12 cells. J. Cell Biol..

[23] Sajjadi S.E., Shokoohinia Y., Mehramiri P. (2013). Isolation and characterization of steroids, phthalide and essential oil of the fruits of *Kelussia odoratissima* Mozaff., an endemic mountain celery. Res. Pharm. Sci..

[24] Chaturvedula V.S., Prakash I. (2012). Isolation of stigmasterol and β-sitosterol from the dichloromethane extract of *Rubus suavissimus*. Int. Curr. Pharm. J..

[25] Luhata L.P., Munkombwe N.M. (2015). Isolation and characterization of stigmasterol and β-sitosterol from *Odontonema Strictum* (Acanthaceae). J. Innov. Pharm. Biol. Sci..

[26] Jabeur H., Zribi A., Makni J., Rebai A., Abdelhedi R., Bouaziz M. (2014). Detection of chemlali extra-virgin olive oil adulteration mixed with soybean oil, corn oil, and sunflower oil by using GC and HPLC. J. Agric. Food Chem..

[27] Valitova J.N., Sulkarnayeva A.G., Minibayeva F.V. (2016). Plant sterols: Diversity, biosynthesis, and physiological functions. Biochemistry (Moscow).

[28] Singh B., Singh J.P., Kaur A., Singh N. (2016). Bioactive compounds in banana and their associated health benefits—A review. Food Chem..

[29] Patel S., Rauf A. (2017). Edible seed from Cucurbitaceae family as potential functional foods: Immense promises, few concerns. Biomed. Pharmacother..

[30] Lee Y.M., Haastert B., Scherbaum W., Hauner H. (2003). A phytosterol-enriched spread improves the lipid profile of subjects with type 2 diabetes mellitus. A randomized controlled trial under free-living conditions. Eur. J. Nutr..

[31] Tatematsu K., Fuma S.Y., Nagase T., Ichikawa Y., Fujii Y., Okuyama H. (2004). Factors other than phytosterols in some vegetable oils affect the survival of SHRSP rats. Food Chem. Toxicol..

[32] Al-Okbi S.Y., Mohamed D.A., Hamed T.E., Esmail R.S., Donya S.M. (2014). Prevention of renal dysfunction by nutraceuticals prepared from oil rich plant foods. Asian Pac. J. Trop. Biomed..

[33] Almeida C.A.S., Baggio S.R., Mariutti L.R.B., Bragagnolo N. (2020). One-step rapid extraction of phytosterols from vegetable oils. Food Res. Int..

[34] Bin Sayeed M.S., Ameen S.S. (2015). Beta-sitosterol: A promising but orphan nutraceutical to fight against cancer. Nutr. Cancer.

[35] Muti P., Awad A.B., Schünemann H., Fink C.S., Hovey K., Freudenheim J.L., Wu Y.W., Bellati C., Pala V., Berrino F. (2003). A plant food-based diet modifies the serum β-sitosterol concentration in hyperandrogenic postmenopausal women. J. Nutr..

[36] Awad A.B., Roy R., Fink C.S. (2003). β-Sitosterol, a plant sterol, induces apoptosis and activates key caspases in MDA-MB-231 human breast cancer cells. Oncol. Rep..

[37] Ayaz M., Junaid M., Ullah F., Subhan F., Sadiq A., Ali G., Ovais M., Shahid M., Ahmad A., Wadood A. (2017). Anti-Alzheimer’s studies on β-sitosterol isolated from *Polygonum hydropiper* L.. Front. Pharmacol..

[38] Shi C., Liu J., Wu F., Zhu X., Yew D.T., Xu J. (2011). β-Sitosterol inhibits high cholesterol-induced platelet β-amyloid release. J. Bioenerg. Biomembr..

[39] Vanmierlo T., Weingärtner O., van der Pol S., Husche C., Kerksiek A., Friedrichs S., Sijbrands E., Steinbusch H., Grimm M., Hartmann T. (2012). Dietary intake of plant sterols stably increases plant sterol levels in the murine brain. J. Lipid Res..

