# Supplementary material for: Neurite Outgrowth-Promoting Activity of Compounds in PC12 Cells from Sunflower Seeds
Source: Molecules. 2020 Oct 16;25(20):4748. doi: 10.3390/molecules25204748 (PMC7587564; doi:10.3390/molecules25204748)
Supplement: Supplementary file 1 [file molecules-25-04748-s001.pdf]

Supplementary Materials

Neurite outgrowth-promoting activity of compounds in PC12 cells from sunflower seeds

Takeru Koga [1], Takaiku Sakamoto [2], Eiji Sakuradani [2] and Akihiro Tai [2]

[1] *Graduate School of Advanced Technology and Science, Tokushima University, 2-1 Minamijosanjima-cho, Tokushima 770-8506, Japan*

[2] *Graduate School of Technology, Industrial and Social Sciences, Tokushima University, 2-1 Minamijosanjima-cho, Tokushima 770-8513, Japan*

Contents:

Figure S1. Neurite outgrowth-promoting activity of sunflower seed extract in the presence of NGF in PC12 cells.

Figure S2. Neurite outgrowth-promoting activity of sunflower seed extract in the presence of Bt_2cAMP in PC12 cells.

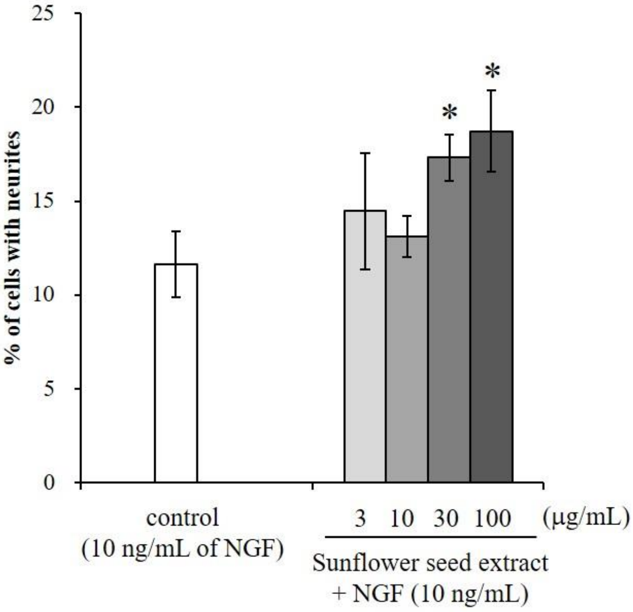

Figure S1. Neurite outgrowth-promoting activity of sunflower seed extract in the presence of NGF in PC12 cells. Sunflower seed extract was diluted with the basal culture medium. PC12 cells were plated at 2.0 x 10^3 cells/well and cultured with the samples at 3-100 μg/mL in the presence of 10 ng/mL of NGF. The extent of neurite outgrowth was measured at 48 hours and is expressed as the mean percentage of 300-400 cells. The data represent means ± SD from three independent experiments. *$p<0.05$ (Dunnett's test) as compared with control (10 ng/mL of NGF only).

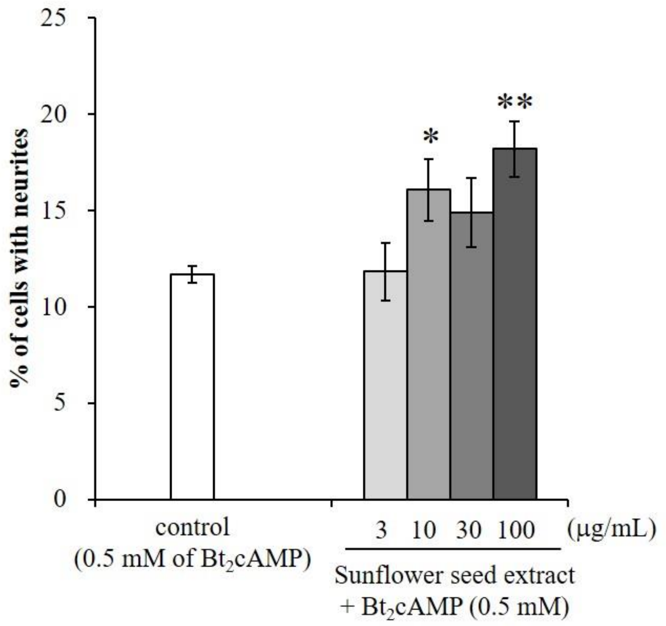

Figure S2. Neurite outgrowth-promoting activity of sunflower seed extract in the presence of Bt_2cAMP in PC12 cells. Sunflower seed extract was diluted with the basal culture medium. PC12 cells were plated at 4.0×10^3 cells/well and cultured with the samples at 3-100 μg/mL in the presence of 0.5 mM of Bt_2cAMP. The extent of neurite outgrowth was measured at 24 hours and is expressed as the mean percentage of 300-400 cells. The data represent means ± SD from three independent experiments. *p<0.05, **p<0.01 (Dunnett's test) as compared with control (0.5 mM of Bt_2cAMP only).
